# Infinitesimal Periangular Pterygomasseteric Transectioning Approach for the Base Fractures of the Mandibular Condyle: A Technical Note and Quality of Life Outcome

**DOI:** 10.7759/cureus.37908

**Published:** 2023-04-20

**Authors:** Darpan Bhargava, Sivakumar Beena, Preeti G Bhargava, V Vidya Devi

**Affiliations:** 1 Department of Oral and Maxillofacial Surgery, TMJ Consultancy Services, Bhopal, IND; 2 Department of Oral and Maxillofacial Surgery, Kamineni Institute of Dental Sciences, Narketpally, IND

**Keywords:** tmj surgery, temporomandibular joint, ippta, periangular incision, maxillofacial, oral surgery, mandible, fracture fixation, fracture reduction, mandibular condyle

## Abstract

Purpose

Infinitesimal Periangular Pterygomasseteric Transectioning Approach (IPPTA) is a minimal access surgical approach utilized for the management of base fractures of the mandibular condyle. The aim of the study was to evaluate and report the long-term post-operative functional outcome using this surgical access approach.

Materials and method

A prospective clinical study involving 20 patients was undertaken to evaluate the post-operative functional and aesthetic outcome for the patients that underwent surgery for base fractures of the mandibular condyle using IPPTA. The various parameters assessed were wound healing, marginal mandibular nerve injury, diet intake, mandibular function, and any other complications at twelfth post-operative month.

Results

IPPTA provided adequate exposure to the condylar base fracture for open reduction and internal fixation (ORIF) and was found to have an uneventful post-operative recovery phase in terms of functional and aesthetic outcomes.

Conclusion

IPPTA involves utilizing a smaller incision and provides adequate exposure to the condylar base region for ORIF to establish a satisfactory form and function with a predictable outcome.

## Introduction

The mandibular condyle and its temporal articulation have complex surgical anatomy, which pose a challenge to performing open joint surgeries owing to the proximity to vital anatomical structures. This necessitates the need to perform minimally invasive approaches to gain access to the temporomandibular joint to prevent trauma to the surrounding tissues. The condylar neck is a region with osseous trajectories organised in a manner that makes this area susceptible to fracture when a substantial indirect or direct impact is transmitted. As a protective phenomenon, the mandibular condyle and the region of the neck intercept the transmission of force to the skull base, which may even lead to fracture of the mandibular condyle at various anatomical locations depending on the magnitude and direction of the impact [1]. To achieve a satisfactory functional and aesthetic outcome, where indicated, open reduction with internal fixation (ORIF) for condylar fractures should be carried out to achieve anatomic reduction with an objective to improve the post-operative quality of life (QoL) [1-3].

There are several approaches documented in the literature to accomplish access to various areas of the condyle, including the condylar base, neck, and head [4-6]. In order to achieve safe surgical access with periangular incision, a minimal invasive surgery was proposed by the authors for the condylar base fractures and was described as the Infinitesimal Peri-angular Pterygomasseteric Transectioning Approach (IPPTA) for ORIF [7]. The present study, is designed to assess the post-operative outcome following restoration of form and function in patients who underwent ORIF for condylar base fractures using IPPTA.

## Materials and methods

A prospective clinical follow-up study was undertaken involving 20 patient volunteers who underwent surgery for condylar base fractures using IPPTA. All the patients in the study were allowed to withdraw from the study at any point without any negative consequences. An informed consent was obtained from all the patients. All the patients underwent ORIF for condylar base fracture using IPPTA for the surgical access by a single qualified operator. This study was a follow up of the patient cohort operated utilizing IPPTA proposed by Bhargava et al. [7]. The patients were followed up for a period of one year to assess the post-operative outcome in terms of restored form and function following ORIF using IPPTA. A single trained research assistant assessed parameters that included: marginal mandibular nerve weakness, wound healing at the incision site, diet intake, mandibular movements, pain associated with mandibular movements, and other complications, if any.

The primary inclusion criteria for the study were: patients in the age group between 18 and 55 years diagnosed with unilateral isolated base fracture of the mandibular condyle in accordance with the Strasbourg Osteosynthesis Study Group (SORG) Classification [8] requiring ORIF. Only patients compliant for a 12-month post-operative followup for the study were included. Exclusion criteria included patient who underwent surgery for diacapitular and condylar neck fractures or any condylar base fracture associated with concomitant other facial fractures requiring additional access incisions. The patients with systemic chronic diseases or medical conditions which alter the normal healing, were also excluded from the study.

Technique and surgical procedure

The surgical procedures were carried under strict aseptic conditions by a single qualified maxillofacial surgical specialist, to eliminate the operator centric bias. All the procedures were undertaken under general anaesthesia, utilizing naso-endotracheal intubation.

**Figure 1:**
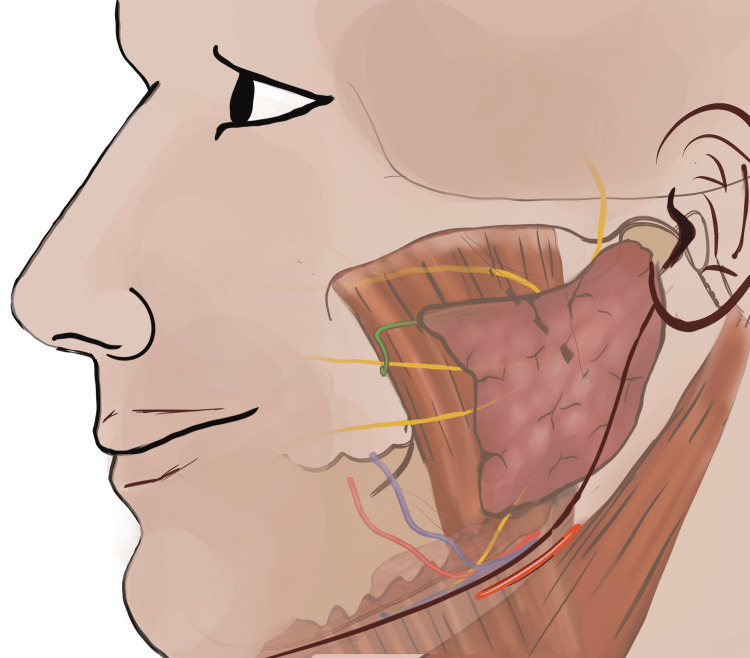
Location of the skin incision to access the base of the mandibular condyle utilizing IPPTA IPPTA: Infinitesimal Peri-angular Pterygomasseteric Transectioning Approach (This figure is an original medical artwork supported by TMJ Foundation describing the steps for Infinitesimal Peri-angular Pterygomasseteric Transectioning Approach).

For the ORIF, minimal access to the condylar base region was attained utilizing IPPTA. For hydro dissection and local haemostasis, the surgical site was infiltrated with a local anaesthetic (LA) solution containing 2% lidocaine with 1:200000 epinephrine (Lox, Neon, India). The peri-angular incision was placed approximately 1.5 cm inferior from the palpable bony angle (gonion) of the mandible (Figure 1). The length of the incision was limited to 1.5 to 2.0 cm following the curvature of the mandibular angle. The length of the incision was determined for the individual patient, based on the build (skinny patients - 1.5 cm, obese patients with bulky neck - 2.0 cm).

The important surgical landmarks that require identification during inward, superior, and upward dissection include platysma, superficial layer of deep cervical fascia, masseter muscle, pterygomasseteric sling, and the tail of the parotid salivary gland. The palpable landmarks include gonion, anti-gonial notch, sub-mandibular salivary gland, and anterior attachment of masseter at the lower mandibular border for orientation during surgical navigation. The aim of the surgical dissection was to reach the sub-masseteric plane by transectioning the pterygomasseteric sling between the tail of the parotid and anterior masseteric attachment to avoid facial vessels followed by stripping lateral ramal-masseteric fibre attachments along the posterior border of ramus to access condylar base region [7].

**Figure 2:**
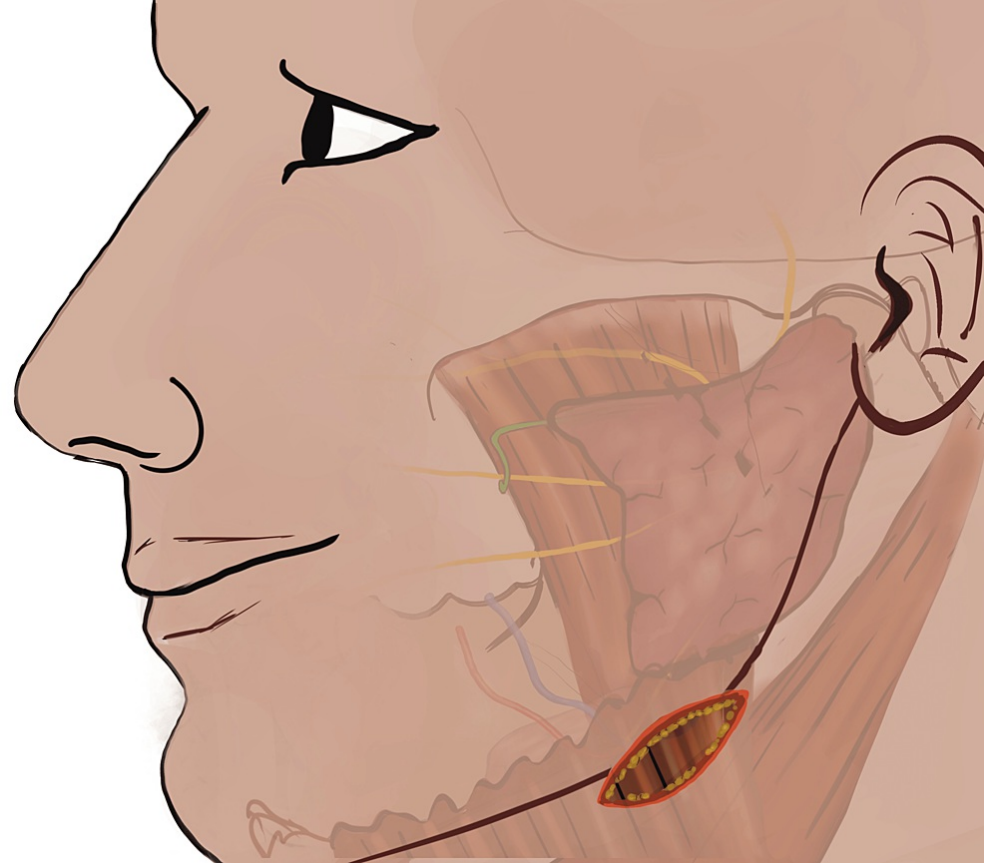
Following the skin incision, the platysmal muscle plane is identified beneath the sub-cutaneous fat. (This figure is an original medical artwork supported by TMJ Foundation describing the steps for Infinitesimal Peri-angular Pterygomasseteric Transectioning Approach).

**Figure 3:**
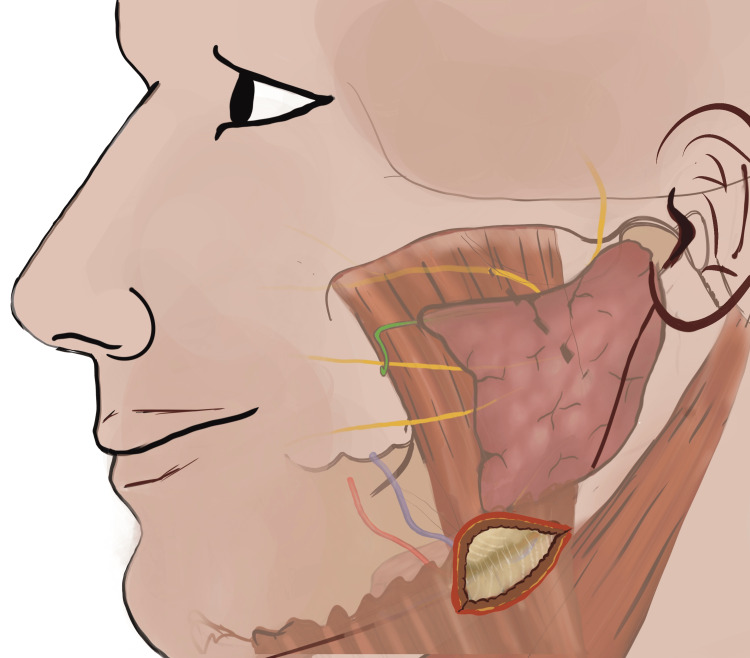
Subplatysmal fascia beneath the platysma muscle plane is identified and dissection proceeds to identify sub fascial structures. (This figure is an original medical artwork supported by TMJ Foundation describing the steps for Infinitesimal Peri-angular Pterygomasseteric Transectioning Approach).

After placing the curvilinear incision perpendicular to the skin, sub-cutaneous fat tissue plane was visualized. From this sub-cutaneous fat tissue plane the dissection was directed inward and superiorly with each surgical plane. After incising the sub-cutaneous tissue identified as yellowish fatty plane, platysma was identified (Figure 2) and dissected carefully preserving the underlying cervical fascia of this region (Figure 3) [7].

**Figure 4:**
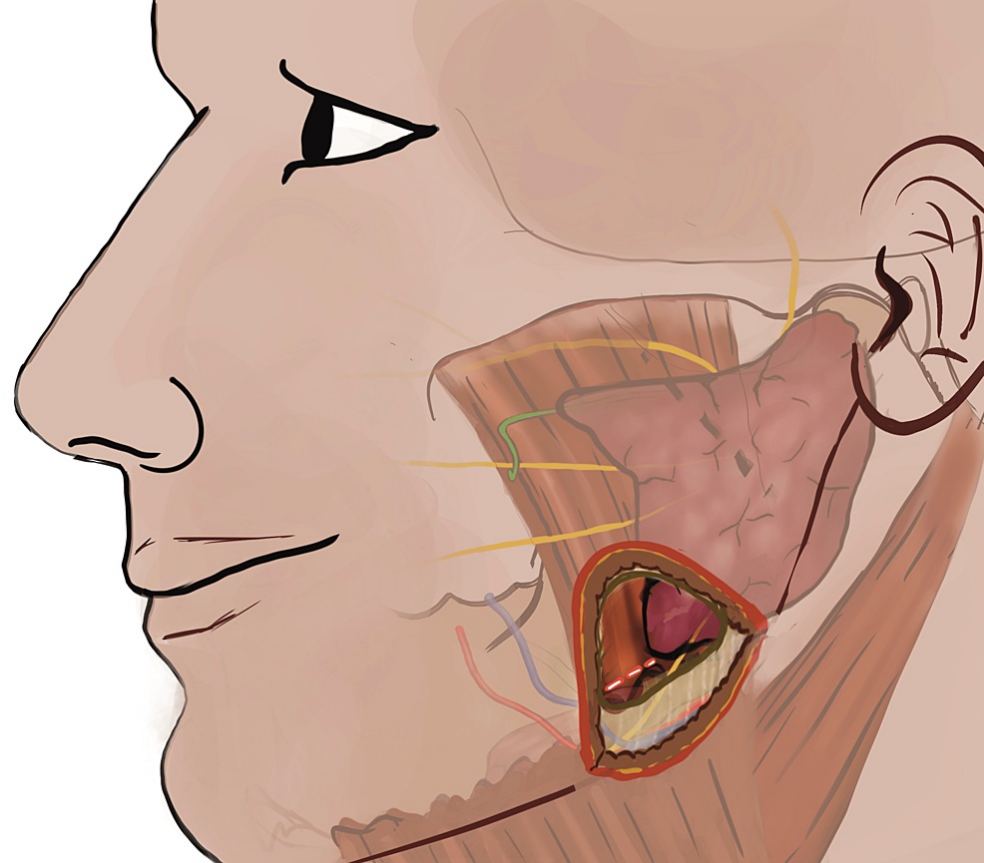
Identification of the angle with the overlying pterygomasseteric sling between the posterior tail of the parotid and anterior masseteric attachment at the inferior border of the mandible. In the sub-fascial plane, the angle with the overlying pterygomasseteric sling between the posterior tail of the parotid and anterior masseteric attachment at the inferior border of the mandible is identified. With adequate retraction of the vital structures in the vicinity, the pterygomasseteric sling is transected and the masseter is stripped in the posterior-cranial direction. (This figure is an original medical artwork supported by TMJ Foundation describing the steps for Infinitesimal Peri-angular Pterygomasseteric Transectioning Approach).

The fascia was incised with close monitoring for any evidence of twitch in the lower lip or surrounding tissues, while using a fine (needle) point cautery tip, as in this region marginal mandibular nerve is immediately above or adherent to the fascia. Once the fascial plane was incised, the pterygomasseteric sling (Figure 4) was identified between the tail of the parotid posteriorly and anterior masseteric attachment at the lower border of mandible.

**Figure 5:**
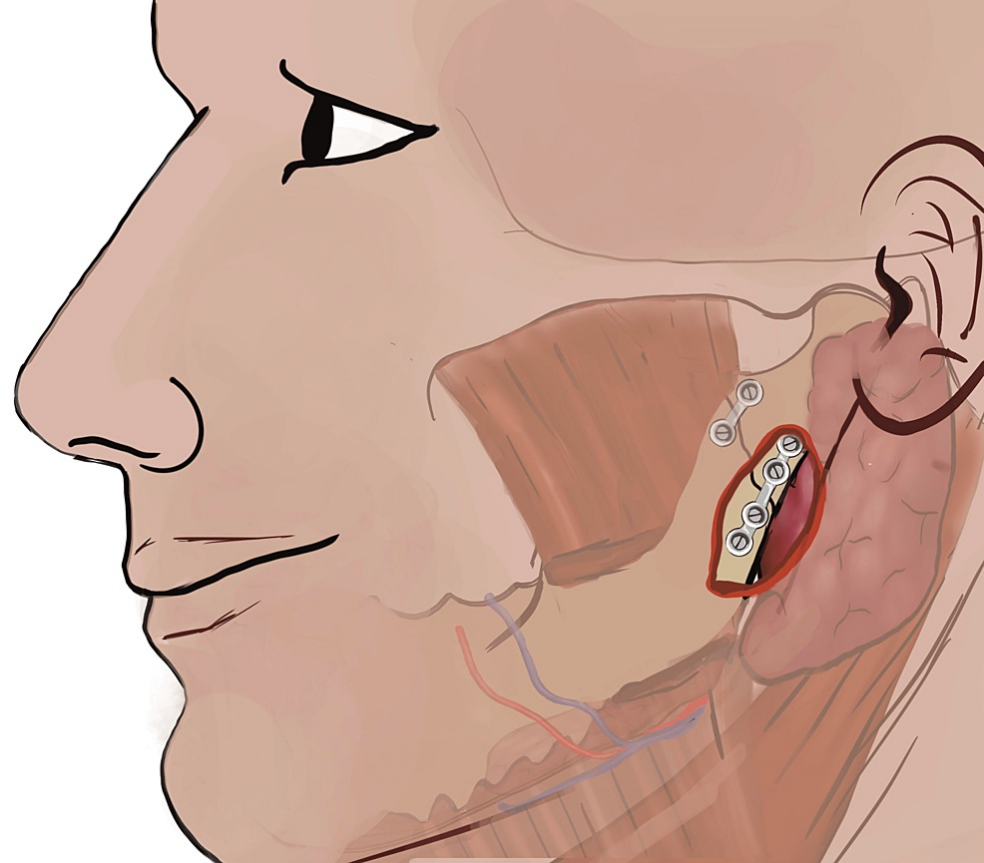
Access to the base of the mandibular condyle utilizing the IPPTA for reduction and fixation using maxillofacial miniplates and screws. Access to the base of the mandibular condyle utilizing the Infinitesimal Peri-angular Pterygomasseteric Transectioning Approach (IPPTA) for reduction and fixation using maxillofacial miniplates and screws. Prototype base fracture of the mandibular condyle for the study was defined in accordance with the Strasbourg Osteosynthesis Study Group (SORG) where the fracture line runs behind the mandibular foramen and more than 50% below the perpendicular line through the sigmoid notch to the tangent of ramus. (This figure is an original medical artwork supported by TMJ Foundation describing the steps for Infinitesimal Peri-angular Pterygomasseteric Transectioning Approach).

The pterygomasseteric sling was transected preserving the parotid posteriorly and keeping transection short of anterior masseteric attachment at the lower border of mandible. After transecting the sling, dissection remains in the sub-masseteric plane, separating the muscular attachments from lateral and posterior ramal border superiorly, till condylar base was exposed. ORIF of the condylar base was performed using titanium miniplates and screws (Figure 5) along with achieving satisfactory occlusion assisted with intraoperative maxillo-mandibular fixation (MMF) [7].

This approach gave a satisfactory surgical field, without any major vascular bleed during dissection. Adequate retraction and planned exposure provide a satisfactory field for surgery for condylar base reduction and fixation following principles of mini-plate functional osteosynthesis. After the acceptable reduction and fracture fixation, haemostasis was achieved, and wound was irrigated with copious normal saline. The closure was done in layers re-approximating masseteric sling, fascia and platysma, subcutaneous layer, and the skin. Appropriate pressure dressing using elastic adhesive bandage (elastoplast/dynaplast) was applied for up to third post-operative day, which sufficed for all cases without the need for a surgical drain, when using this surgical incision. As a standard protocol following implanting the hardware, injection cefotaxime 1gm twice a day (BD), diclofenac sodium 75mg/1ml (aqueous) BD, metronidazole 500mg/100ml thrice a day (TDS), pantoprazole 40mg once a day (OD) was administered intravenously (IV) for up to 3rd post-operative day followed by tablet cefixime 200mg BD, diclofenac (50mg)/acetaminophen (325mg) combination BD and pantoprazole 40mg OD per oral up to the 5th post-operative day [7]. All the patients were advised soft diet for one week in the post-operative period.

Patients were followed up in the immediate post-operative period for the requirement of maxillo-mandibular fixation (MMF) and the data was recorded for the study. At 12th month follow-up, the patients were evaluated for marginal mandibular nerve deficit, wound healing at the incision site, diet intake, mandibular function, and other complications, if any [7].

Data collection for the study

The patients were followed up for a period of one year by a single trained research assistant. The parameters assessed at the 12th month follow-up were recovery from marginal mandibular nerve weakness (if any), wound healing at incision site, diet intake, mandibular movements (voluntary/non-assisted), and any other complications (if any). Marginal mandibular weakness recovery was visually evaluated by checking for symmetry and hypomobility of the lower lip. Wound healing was assessed by visual evaluation of the incision line, presence of signs of infection and by evaluating the sensation in the surrounding skin. Diet intake was assessed using a modified Likert scale [9]. The patients were provided with a format containing the modified Likert scale along with instructions (Table 1). It was also explained verbally to the patient. Mandibular movements, pain associated with mandibular movements was assessed using Helkimo’s Clinical Dysfunction Index [10].

**Table 1:** MLTPS for mandibular trauma Modified Likert Type Psychometric Scale (MLTPS) for mandibular trauma to assess dietary intake following ORIF of the base fractures of the temporomandibular joint in the post-operative period.

MLTPS Score	Interpretation
1	Ability to chew food of any consistency
2	
3	
4	Can chew solid foods with minimal discomfort
5	
6	
7	
8	Difficulty in chewing solid foods. Can consume semi-solid foods with minimal discomfort
9	
10	Only liquids

## Results

**Figure 6:**
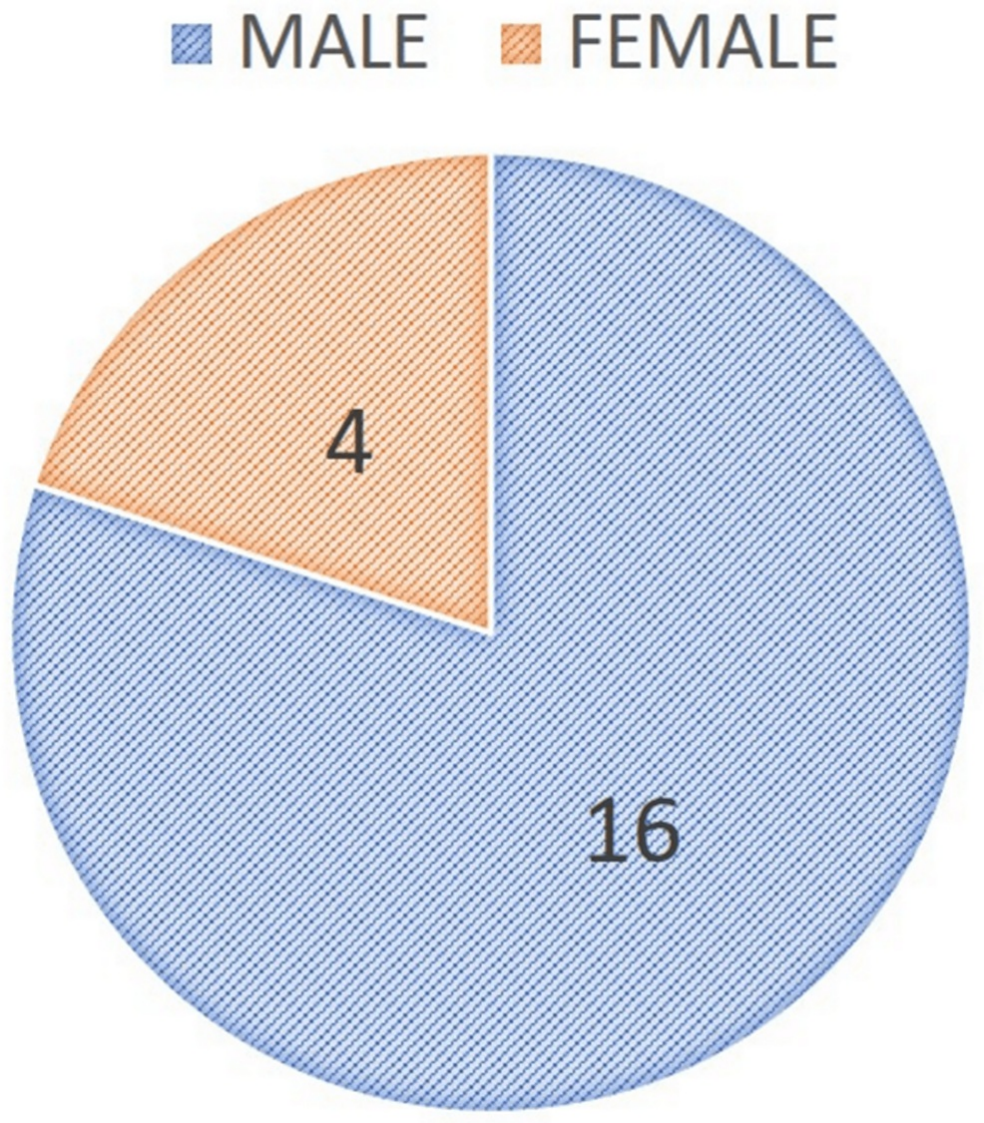
Gender variance in the study subjects with condylar base fractures operated utilizing IPPTA. IPPTA: Infinitesimal Peri-angular Pterygomasseteric Transectioning Approach

The study included a total of 20 patients, n = 20 (100%). Among the 20 patients, n = 16 (80%) were males and n = 4 (20%) were females. The mean age of the study patients was 31±8.53 years (Min 18; Max 55). The long-term evaluation was done at the 12th month follow-up (Figure 6). The visual inspection to evaluate the lower lip symmetry and hypo-mobility to rule out marginal mandibular nerve injury revealed that all the patients, n = 20 (100%) had no weakness or drooping of the lower lip on smiling or blowing of the mouth. All the study population patients had no evidence of deficit in the nerve function at the 12th post-operative month with complete restoration of lip symmetry and function. 

All the patients, n = 20 (100%) showed satisfactory wound healing at the incision site with minimally visible scar, n = 3 (15%) patients complained of hardness at the incision site with reported tenderness on palpation. This did not affect the patient’s jaw function, as reported by the patients. Restoration of diet intake was assessed by using the modified Likert scale. Among 20 patients, n = 14 (70%), n = 4 (20%), and n = 2 (10%) patients gave a score of one, two and three respectively, indicating that they were able to chew food of any consistency, at 12th post operative month (Table 1).

The mandibular movements and the pain associated with mandibular movement was assessed using Helkimo’s Clinical Dysfunction Index. Majority of the patients demonstrated no adverse symptoms associated with the temporomandibular joint (TMJ) (Table 2). Two patients (10%) exhibited impaired TMJ function with evidence of click. Two (10%) patients (self) reported impaired range of motion (ROM) of which n = 1 patient did not receive MMF and one patient received MMF for 21 days in the post-operative period. Among the study population, n = 1 (5%) patient had impairment of mandibular ROM, clinical evidence of click and tenderness on palpation. None of the patients reported any significant adverse effect or complication that affected normal mandibular function.

**Table 2:** Impairment in mandibular movement, function and pain assessed by Helkimo’s Clinical Dysfunction Index (at 12th month follow-up after ORIF utilizing IPPTA). TMJ: Temporomandibular joint; ORIF: Open reduction and internal fixation; IPPTA: Infinitesimal Peri-angular Pterygomasseteric Transectioning Approach *one of the patients with self-reported impaired ROM and the presence of click-on examination reported joint pain on function.

Score	Impaired range of motion (ROM)	Impaired TMJ function	Muscle pain	TMJ pain	Pain on mandibular movement at the incision site
0 (Absence of symptoms)	18 (90%)	18 (90%)	20 (100%)	19 (95%)	20 (100%)
1 (Mild symptoms)	2* (10%)	2* (10%)	0 (0%)	1* (5%)	0 (0%)
5 (Acute symptoms)	0 (0%)	0 (0%)	0 (0%)	0 (0%)	0 (0%)

## Discussion

**Figure 7:**
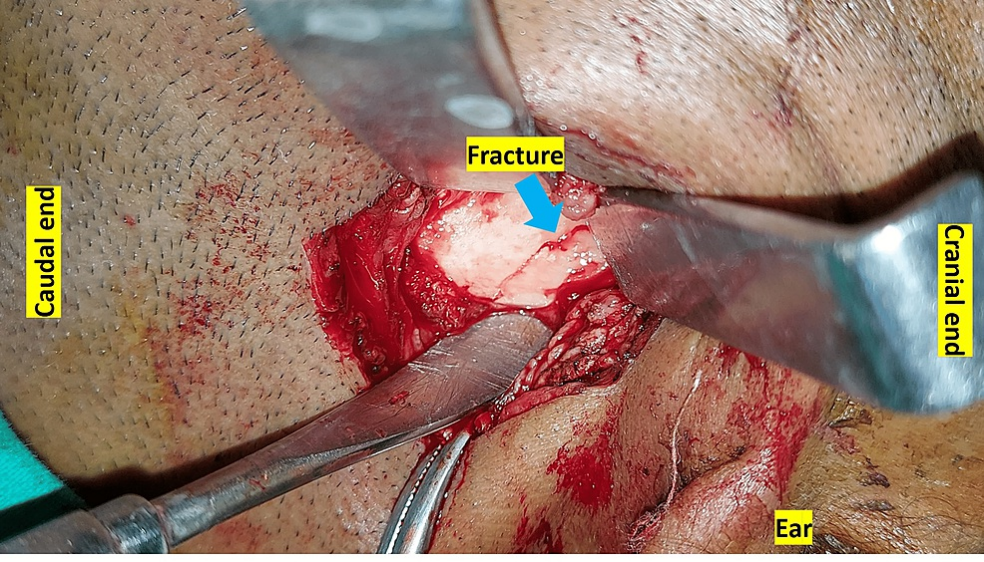
Surgical exposure of the condylar base region utilizing IPPTA IPPTA: Infinitesimal Peri-angular Pterygomasseteric Transectioning Approach

In general, with the comprehensive understanding of the surgical anatomy, the minimal access surgery is being adopted for the surgical procedures, where indicated. Infinitesimal Peri-angular Pterygomasseteric Transectioning Approach (IPPTA) is a technical modification as a minimal access to existing peri-angular approach to the lateral ramus and mandibular condylar base region. Among the various surgical approaches to the mandibular condyle, peri-angular incision is categorized as high sub-mandibular or the modified limited access high Risdon’s approach [11]. The tissue dissection for this approach lies in between the buccal and marginal mandibular nerve and is commonly used for exposing the lateral mandibular ramus up to the condylar base. The IPPTA has been advocated to expose the condylar base fracture for its reduction and fixation [7]. This modified approach involves a small curvilinear incision with mandibular angle as the landmark and dissection along the tissue planes in the direction parallel to the nerve fibres limiting the probability of damage to the surrounding vital structures. The advantages of IPPTA include adequate condylar base exposure, avascular surgical plane, optimal access to the surgical site (Figure 7) and early functional recovery. The operator feedback, clinical follow up, and patient evaluation using Modified Likert Type Psychometric Scale (MLTPS for Mandibular Trauma) and Helkimo’s Clinical Dysfunction Index at twelfth month after ORIF utilizing IPPTA, demonstrate a favourable outcome with minimal tissue trauma. The reported acceptable VAS pain scores and satisfactory ROM with TMJ function, favour the use of minimal access to condylar base utilizing IPPTA. The study reports negligible morbidity and uneventful recovery at the 12th month follow up period in majority of the patients subjected to IPPTA. 

Ellis et al. evaluated the surgical complications with open treatment of mandibular condyle fractures. They observed that there were few intra-operative and post-operative complications in patients undergoing open surgery for trauma to the mandibular condyle. Post-operative complications included infection, facial nerve palsy, auriculotemporal nerve dysfunction, Frey’s syndrome, and an unsightly scar. They emphasized that several factors should be considered to opt for either an open or closed method to treat condylar base fractures. In case of open reduction and fixation of condylar fracture, minimal damage to the surrounding vital structures should be considered to avoid complications [12].

García-Guerrero et al. assessed the complications for both open and closed reduction of mandibular condylar fractures and observed that complications associated with the technique utilized are usually minimal and infrequent suggesting that a proper knowledge of anatomy is essential for the operating surgeon to minimize iatrogenic damage. They observed that complications associated with open reduction were quite less as compared to the result and complications for closed reduction causing delayed immobilisation and functional limitation. Appropriate diagnosis and case selection should be considered to perform ORIF for condylar fracture thereby minimising morbidity and incidence of facial nerve damage [13,14].

The limitation of this study remains in the smaller sample size which further require validation of results with larger multicentric randomised controlled trials. The development of joint dysfunction would require evaluation with a longer follow up period, although it may not influence the efficacy of the surgical approach utilized. Further, as the directions to future research, Infinitesimal Peri-angular Pterygomasseteric Transectioning Approach may be compared with other conventionally utilized surgical approaches for the base fractures of the temporomandibular joint.

## Conclusions

It is crucial to weigh the benefits over risks in utilizing various treatment strategies to perform an open reduction for condylar fractures. Minimally invasive approaches have a specific indication that should be adhered to. Among various approaches available for the ORIF of the condylar fractures, IPPTA provides adequate surgical access to the condylar base with minimal morbidity and favourable post-operative outcomes. IPPTA involves utilizing a smaller incision and provides adequate exposure to the condylar base region for ORIF to establish a satisfactory form and function with a predictable outcome.
